# Sensory Function and Chronic Pain in Multiple Sclerosis

**DOI:** 10.1155/2018/1924174

**Published:** 2018-04-23

**Authors:** Rogier J. Scherder, Neeltje Kant, Evelien T. Wolf, Bas C. M. Pijnenburg, Erik J. A. Scherder

**Affiliations:** ^1^Department of Clinical Neuropsychology, Vrije Universiteit, Amsterdam, Netherlands; ^2^Department of Neuropsychology, Reade, Amsterdam, Netherlands; ^3^Acibadem International Medical Center, Amsterdam, Netherlands

## Abstract

**Objective:**

To examine whether hypoesthesia and chronic pain are related in patients with MS.

**Methods:**

Sixty-seven MS patients with pain and 80 persons without MS were included. Sensory functioning was tested by bedside neurological examination. Touch, joint position (dorsal column-medial lemniscus pathway), temperature sense, and pain (spinothalamic tract) were tested. Pain intensity was measured by the Colored Analogue Scale (CAS Intensity) and the Faces Pain Scale (FPS); pain affect was also measured by CAS Affect and Number of Words Chosen-Affective (NWC-A). Mood was assessed with the SCL-90 anxiety and depression subscales and the Beck Depression Inventory (BDI).

**Results:**

A significant negative relationship was found between pain intensity and the function of the dorsal column-medial lemniscal pathway, but not with the spinothalamic tract.

**Conclusion:**

In addition to the already known relation between hyperesthesia and pain, hypoesthesia for touch and joint position also seems to be related to chronic pain in MS patients.

## 1. Introduction

Chronic pain is a common symptom in multiple sclerosis (MS) [1]. Between 29 and 86 percent of all MS patients suffer from chronic pain [2]. This pain has been described in MS as both nociceptive and neuropathic [1, 3]. Nociceptive pain occurs when nociceptors are activated in response, for example, to tissue damage [4]. In MS, nociceptive pain can be provoked by abnormalities in the musculoskeletal system, for example, spasms [1]. Neuropathic pain may include both central and peripheral neuropathic pain and can be caused by lesions in the brain or spinal cord [5, 6].

A lesion in the brain or spinal cord may express itself in sensory disturbances. It is well known that MS is characterized by sensory disturbances reflected in both hyper- and hypoesthesia [5]. Hyperesthesia is often expressed in allodynia, a painful response to a nonpainful stimulus [7]. Allodynia is also a characteristic of central neuropathic pain [8]. There is thus a direct relationship between sensory disturbances, like allodynia, and pain, that is, central neuropathic pain, in MS. However, patients with chronic pain conditions (e.g., osteoarthritis, musculoskeletal pain, and peripheral and central neuropathy) may show a *decline* in sensory functions, for example, hypoesthesia to touch [9–11]. The question arises as to whether a decline in sensory functions and chronic pain is also interrelated phenomena in MS. Support for a relationship between hypoesthesia and chronic pain emerges from a study investigating sensory functions of MS patients with and without chronic pain [12]. Compared with MS patients without pain, the group of MS patients with pain, irrespective of its nature, showed a decreased sensitivity for, among others, vibration, joint position, and touch assessed by bedside sensory testing. In that study, the functioning of the dorsal column-medial lemniscal pathway was assessed using vibration, joint position, and touch and the functioning of the spinothalamic pathway using pain and temperature [12]. However, the results of that remain obscure as to whether hypoesthesia was related to pain.

Compared with the role of the spinothalamic pathway in pain, little is known about the role of the dorsal column-medial lemniscal pathway in chronic pain in MS [13]. In examining the role of both the spinothalamic pathway and the dorsal column-medial lemniscal pathway in the relationship between sensory functions and pain, a distinction between pain intensity and pain affect was made in the present study. Such a distinction is known in pain research. A brain region that plays a crucial role in the processing of pain intensity is the somatosensory cortex [14], which is the target area of the dorsal column-medial lemniscal pathway [15]. Pain affect is processed by the spinothalamic tract, projecting to the prefrontal cortex, among others [14]. In other words, distinguishing between pain intensity and pain affect may provide more insight into the functioning of the dorsal column-medial lemniscal pathway and the spinothalamic tract.

The cumulative goal of the present study was thus to examine a possible relationship between chronic pain intensity and affect, and a decline in sensory functioning in patients with MS. Such a relationship may be clinically relevant as it implies that the presence of chronic pain in MS may be reflected by both hypo- and hyperesthesia. This study hypothesizes that, depending on the types of sensory dysfunction, that is, temperature, pain, light touch, and position sense, hypoesthesia may also be indicative of chronic pain intensity, pain affect, or both.

## 2. Materials and Methods

### 2.1. Study Design

The present cross-sectional study was part of a larger study examining the relationship between pain and cognition in patients with multiple sclerosis [16, 17].

### 2.2. Participants

From the larger study, we included in the present study 67 MS patients who suffered from chronic pain. MS patients were recruited from a center, specialized in MS and other neurodegenerative disorders in the Netherlands, or enrolled from the personal environment of the researchers. In each case, an official diagnosis of MS was made by a neurologist, according to the criteria of Poser or McDonald criteria [18, 19]. We also included 80 persons without MS (control group). Well-instructed and trained medical and psychology students tested the MS patients and the controls.

### 2.3. Education

Both the MS patients and the control participants were screened for education. Education was divided into five categories: elementary school not finished (score = 1), elementary school (score = 2), lower secondary school (score = 3), higher secondary school (score = 4), and higher vocational training for 18+/university (score = 5).

### 2.4. Chronic Painful Conditions

Both groups suffered from arthrosis/rheumatoid arthritis, musculoskeletal disorders (e.g. neck-shoulder pain), migraine, osteoporosis, and peripheral neuropathic pain. Peripheral neuropathic pain was due to metatarsalgia, carpal tunnel syndrome, low back pain with irradiation, and meralgia paresthetica. Probably, our participants did not suffer from central neuropathic pain reflected in an absence of allodynia. Chronic pain was defined as pain occurred during a period of 3 months or longer [20].

### 2.5. Medication

Within the scope of the present study, we listed analgesics (baclofen, paracetamol, diclofenac, naproxen, ibuprofen, and cannabis) and medication that might be related to sensory disturbances: sedatives (e.g., temazepam), antipsychotics (e.g., Fluanxol), antidepressives (e.g., citalopram), anxiolytics (e.g., Rivotril), and neurological disorders (e.g., epilepsy: Depakine).

### 2.6. Comorbidities

Comorbidities that might cause sensory disturbances, that is, diabetes, transient ischemic attack (TIA), migraine, and epilepsy, were listed (for a full list of comorbidities see [17]).

### 2.7. Exclusion Criteria

Participants (patients and controls) were excluded if they had a history of neoplasms, cerebral traumata, alcoholism, normal pressure hydrocephalus, disorders of the central nervous system other than MS, or disturbances of consciousness.

### 2.8. Informed Consent

The local medical ethical committee gave their approval for the present study (NL 19801.029.07, 2007.211). The patients were asked to give oral and written consent after they had been extensively informed about the aim and procedure of the study. After permission, the neuropsychological, the sensory function, and the pain perception tests were obtained. The patients were able to discontinue their participation at any time during the current study.

### 2.9. Sensory Function

Sensory function was tested by a bedside neurological examination. In particular, it was tested whether the sensory function of the MS patient was normal (score = 2) or decreased (score = 1) on two levels: dorsal column-medial lemniscal function (by testing joint position and touch) and function of the spinothalamic cortical pathway (by testing temperature sense and pain). The classification from Svendsen and colleagues was used to assess the spinothalamic cortical pathway and dorsal column-medial lemniscal pathway [12].

#### 2.9.1. Dorsal Column-Medial Lemniscal Pathway

This pathway mediates fine touch and joint position. Fine touch was tested by applying a cotton wool on the dorsal side of the right and left hand, forearm, and upper arm. Joint position was tested by passively stretching or bending one finger of the patient. The patient was asked which finger has been stretched or bended. Three fingers of each hand were moved. Fine touch or joint position were considered to be disturbed if the participant failed, by giving one or more incorrect answers, to indicate whether they were touched or gave an incorrect answer concerning the position of the joint. During all of these tests the patients kept their eyes closed [21].

#### 2.9.2. Spinothalamic Cortical Pathway

This pathway mediates pain and temperature. Pain was tested by applying a pinprick with a needle with either a sharp or blunt end on the dorsal side of the participant's right and left hand, forearm, and upper arm. Temperature sense was evaluated by making a distinction between warm and cold. The investigator touches the dorsal side of the right and left hand, forearm, and upper arm with a small plastic bottle filled with either cold or hot water. The patient kept their eyes closed during the test. If the participant gave one or more incorrect answers, sensory function was considered to be disturbed [21].

### 2.10. Pain

#### 2.10.1. Colored Analogue Scale (CAS)

Two CAS scales were applied: one measures the intensity of pain (CAS Intensity) and the other measures the affective/emotional components of pain (CAS Affect) [22]. The CAS is a visual analogue scale with a plastic slide, which can be moved by the patient from the bottom (“no pain,” light pink) to the top (“maximum pain,” dark red). To measure the intensity or affective/emotional aspects of pain adequately, a scale from 0 to 10 has been drawn (score: 0 = no pain; score: 10 = severe pain) on the back.

#### 2.10.2. Number of Words Chosen-Affective (NWC-A)

This is the affective part of the McGill Pain Questionnaire (Dutch version) and is composed of 5 groups [23]. Each group consists of 3 affective words, for example, alarming, frightening, and terrifying. The patient was asked to choose one word of each group that levels their pain experience (maximum score: 15).

#### 2.10.3. Faces Pain Scale (FPS)

This scale consists of seven different faces with different expressions. Each face represents an increased feeling of pain [24, 25]. The patient was asked to choose one face that matches their pain experience (score: 0 = no pain; score: 6 = severe pain).

Two pain domains were composed: (1) pain intensity, which is composed of the CAS Intensity and the FPS, and (2) pain affect, which is composed of CAS Affect and the NWCA. The MS patients were asked to indicate their level of pain during the last week. This way of measuring the level of pain is reliable, according to a study of Forouzanfar and colleagues [26].

The extent to which patients were suffering from pain is presented in the Results section.

### 2.11. Mood

As pain may be associated with depression and anxiety [27], three questionnaires were used to assess depression and anxiety: the Beck Depression Inventory (BDI) (minimum score = 0; maximum score = 63) [28, 29], the SCL-90 anxiety subscale (minimum score = 0; maximum score = 40) [30], and the SCL-90 depression subscale (minimum score = 0; maximum score = 52) [31].

The scores of these three scales were first converted into z-scores. Subsequently, a reliability analysis resulted in a Cronbach's alpha of 0.84. Next, we made a composite domain score “mood.”

### 2.12. Procedure

The pain scales (CAS Intensity, CAS Affect, NWC-A, and FPS) and sensory tests (touch, joint position, pinprick, and temperature sense) were administered in one session. First, the pain scales were conducted from the patient, after which the sensory functions were assessed.

### 2.13. Data Analysis

We used the SPSS-PC program for the data analyses. Chi-square tests, *t*-tests, and Mann–Whitney *U* tests were applied to analyze data between groups. As some cells had a low cell count, statistical significance was established by means of Fischer's exact tests (two-tailed). The relationships between chronic pain, the dorsal column-lemniscal pathway, and the spinothalamic tract were analyzed by hierarchical linear regression analyses: one model predicting pain intensity and one predicting pain affect. In the first step, mood was added as a predictor; in the second step, the dorsal column-medial lemniscal pathway; and in the third step, the spinothalamic tract was entered. Adjusted *R*^2^ and *R*^2^ are reported as measures of model fit and Δ*R*^2^ as a measure of the effect of sensory functioning (dorsal column-medial lemniscal pathway and the spinothalamic tract) on pain while controlling for mood. The level of significance was set at *p* < 0.05.

## 3. Results

### 3.1. Demographics

#### 3.1.1. Age

The mean age of the MS patients (M = 51.25 years; SD = 9.84) did not differ significantly from the mean age of the control group (M = 48.79 years; SD = 10.49) (*t* (145) = 1.46; *p*=0.15).

#### 3.1.2. Gender

The distribution of gender within the group of MS patients is as follows: 66.2% women and 33.8% men. Within the control group, the distribution of gender was 66.3% women and 33.8% men (chi square: 0.99, *df* = 1, and *p*=0.99).

#### 3.1.3. Education

The mean level of education of the MS patients is 3.57 (SD = 0.76). The mean level of education of the control group was 3.50 (*t* (141) = 0.55; *p* < 0.59).

### 3.2. Mood

With respect to depression and anxiety, MS patients showed significant higher scores than the controls (for means, standard deviations, and Mann–Whitney *U* tests see Table 1). Consequently, concerning the mood domain, the group with MS patients (mean rank: 91.64) differed significantly from the control group (mean rank: 58.11) (Mann–Whitney *U* test: *Z* = 4.78; *p* < 0.001).

### 3.3. Sensory Function

#### 3.3.1. Dorsal Column-Medial Lemniscal Pathway

23.9% of the MS patients had decreased sensibility to touch. Disturbances in joint position were found in 25.8% of the MS patients. Both sensory functions were significantly more disturbed in MS patients than in the control group (see Table 2 for means, standard deviations, and Mann–Whitney *U* tests).

#### 3.3.2. Spinothalamic Tract

**Figure 1:**
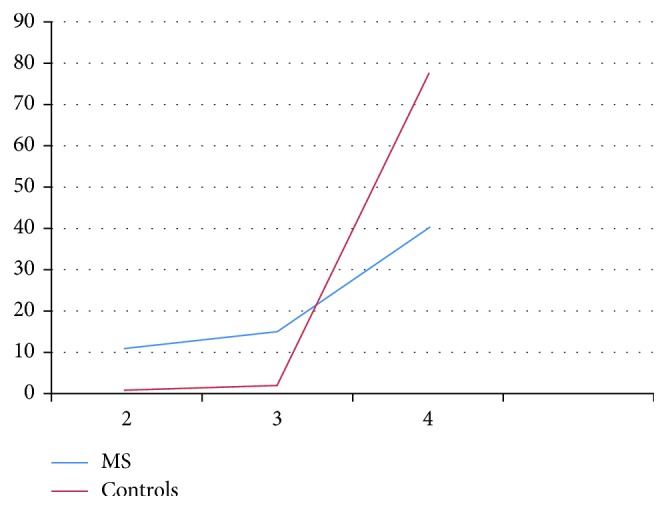
Scores of the patients with multiple sclerosis (MS) and controls on position sense and light touch (dorsal column-medial lemniscal pathway). The lower the score, the more disturbances in sensory functioning.

**Figure 2:**
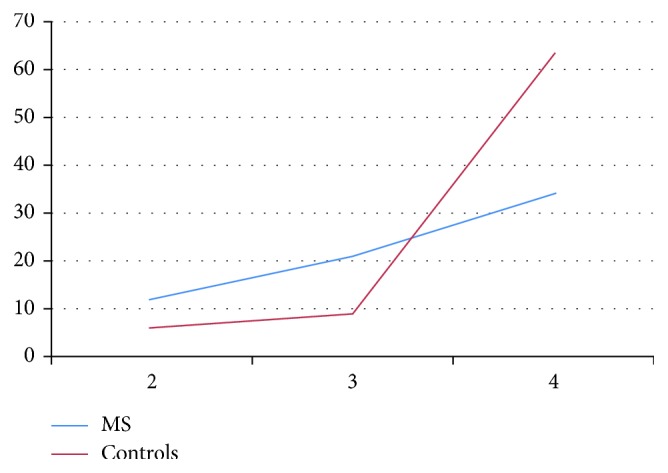
Scores of the patients with multiple sclerosis (MS) and controls on temperature and pain (spinothalamic tract). The lower the score, the more disturbances in sensory functioning.

When pain experience was tested by applying a pinprick, 40.3% had sensory disturbances. Disturbances in temperature were found in 20.9% of the cases. Compared to the control group, MS patients showed significantly more disturbances in both sensory functions (see Table 1 for means, standard deviations, and Mann–Whitney *U* tests; Figures 1 and 2).

### 3.4. Sensory Function in Relation to Comorbidity

The group of MS patients was divided into a group of patients with sensory disturbances (either dorsal column-medial lemniscal pathway or spinothalamic tract or both) and a group of patients without sensory disturbances. We were interested whether, compared to MS patients without sensory disturbances, those with sensory disturbances suffered more from comorbidities that might have caused those sensory disturbances. As can be seen in Table 3, comorbidities such as diabetes mellitus, transient ischemic attack (TIA), migraine, and epilepsy did not differ significantly between both groups. Arteriosclerosis, stroke, and traumatic brain injury appeared not to be present in our patients.

### 3.5. Pain Experience

Data analyses by means of Mann–Whitney *U* tests show that MS patients suffer significantly more from pain intensity and pain affect, compared to controls (for means, standard deviations, and Mann–Whitney *U* tests see Table 4).

### 3.6. Pain Medication

MS patients used significantly more baclofen, paracetamol, and cannabis than the controls (see Table 5 for percentages, chi-square, and Fisher's exact tests).

### 3.7. Sensory Function in Relation to Medication

The use of analgesics and the use of medication prescribed for disorders that might affect sensory functioning (sedatives, antipsychotics, antidepressives, anxiolytics, and medication for neurological disorders, e.g., epilepsy) were examined by means of chi-square test and Fisher's exact test. The results show that concerning analgesics, only cannabis differed significantly between both groups; MS patients with sensory disturbances used significantly more cannabis than those without sensory disturbances. No significant differences between both groups were observed concerning the other medications that might be related to sensory disturbances (see Table 6 for percentages, chi-square test and Fisher's exact test).

### 3.8. Relationship between Pain, Sensory Function, and Mood

#### 3.8.1. The Relationship between Pain Intensity and Sensory Function

The results of a hierarchical regression analysis show a significant relationship between mood and pain intensity, thereby explaining 9% of its variance (Model 1). Adding the functioning of the dorsal column-medial lemniscal pathway explained an additional 10% of variance in pain intensity (Model 2). There was no significant change in explained variance in pain intensity, after adding the function of the spinothalamic tract (Model 3) (Table 7).

#### 3.8.2. The Relationship between Pain Affect and Sensory Function

The results of a hierarchical regression analysis show a significant relationship between mood and pain affect, explaining 9% of the variance in pain affect (Model 1). No significant relationships were found between pain affect and the function of the dorsal column-medial lemniscal pathway (Model 2) and between pain affect and the function of the spinothalamic tract (Model 3) (Table 8).

## 4. Discussion

The goal of the present study was to examine a possible relationship between hypoesthesia and chronic pain in MS patients. The main finding relates to our observation of a significant negative relationship between pain intensity and hyposensitivity concerning light touch and joint position (dorsal column-medial lemniscal pathway) (Table 7). This finding implies that the larger the decline in the perception of light touch and joint position, the higher the pain intensity, and higher the patient suffers. This finding has not previously been described in the MS literature, where there are descriptions of the relationship between hyperesthesia and chronic pain, in particular central neuropathic pain [12, 32]. The lack of a relationship between hypoesthesia and pain affect (Table 8) may be due to the fact that the MS patients indicated less pain affect (NWC-A, CAS Affect) than pain intensity (FPS, CAS Intensity).

The rationale is that chronic pain, known to be present in MS [33], may be due to a dysfunction of the dorsal column-medial lemniscal pathway expressed in hypoesthesia. Alternatively, as has previously been described in the literature, chronic pain such as complex regional pain syndromes and chronic arthropathies may cause hypoesthesia, for example, for touch [34]. One of the underlying mechanisms is that nociceptive activation of the unmyelinated C-fibers may inhibit the processing of nonnociceptive information (e.g., light touch) transmitted by beta fibers, for example, [34]. This might occur at both a spinal and cortical level. Geber and colleagues [34] further suggest that if pain experience decreases through more adequate treatment, the hypoesthesia should diminish.

The main finding yielded related questions. One of these include whether MS patients with sensory disturbances differed from those without sensory disturbances concerning comorbidities that might provoke sensory disturbances. This appeared not to be the case (Table 3). A similar question concerned the use of analgesics and other medications that could be related to sensory disturbances (Table 6). There were no significant differences between MS patients with and without sensory disturbances concerning analgesic medication, except for cannabis. Cannabis is often prescribed for chronic pain and for spasm in MS patients, although its effectiveness is not consistent [35]. Häuser and colleagues [35] also mention possible side effects of cannabis in their review, and disturbances in sensory functioning are not among them. To our knowledge, a negative effect of cannabis on the functioning of the dorsal column-medial lemniscal pathway or on the functioning of the spinothalamic pathway has not yet been reported in literature. Despite the lack of significant differences (except for cannabis) between MS patients with and without sensory disturbances, we analyzed whether there was a relationship between (analgesic) medication and sensory dysfunction; this appeared not to be the case (data not shown).

Compared with controls, the use of baclofen, paracetamol, and cannabis was significantly higher in our MS patients (Table 5). The higher usage of analgesics by MS patients fits the higher level of pain intensity and pain affect in this group, compared with the controls (Table 4).

Our finding concerning the relationship between hypoesthesia and pain intensity in MS patients may be clinically relevant. In cases of hyperesthesia, a patient may respond quite vividly to light touch, for example, due to a considerable increase in pain [36], even when the patient is not able to communicate about pain. However, if the patient hardly responds to light touch, for example, at least two explanations are possible: the first is that the patient experiences the light touch as normal but cannot express his or her feelings clearly enough, and the second is that the patient is actually impaired in processing light touch. Not knowing which of the two is applicable to the patient, the clinician may want to opt for the “worst case scenario,” that is an impairment in processing light touch. In the latter case, the clinician will be motivated to search for the presence of chronic pain.

Our results further show that compared with controls, MS patients had higher scores on scales measuring mood, more specifically depression and anxiety (Table 1). The relatively low mean scores suggest that MS patients suffer from depressive *symptoms* instead of major depressive disorders [37]. We observed a significant relationship between mood and pain intensity and between mood and pain affect in MS patients (Tables 7 and 8). These findings are congruent with previous studies in which depression and pain coincided in MS patients [37]. Feistein and colleagues [37] emphasize the importance of focusing treatment on both symptoms simultaneously [37]. They also state that anxiety disorders may occur even more frequently in MS but do not receive as much attention as depression. Treatment of anxiety is the more important as, untreated, it may further aggravate cognitive decline [37].

The present study has several limitations. Firstly, we were unable to perform quantitative sensory testing (QST) to examine sensory function in MS patients in an objective way. This is because our study was based partly on Svendsen and colleagues' study [12], which examined the sensory functions belonging to the dorsal column-medial lemniscal pathway and the spinothalamic tract by both bedside sensory testing and quantitative sensory testing (QST). Only the bedside sensory testing method appeared to be sensitive for differences in sensory functioning between MS patients with and without pain. MS patients with pain showed a decrease in sensibility in touch, among others [12]. Nevertheless, to assess the perception threshold for touch, vibration, and temperatures [5], QST should be applied in studies examining pain in MS.

In addition to QST, a neurophysiological examination in a study on pain in MS could be conducted, for example, by measuring somatosensory-evoked potentials that are transmitted by thick-myelinated A*β* fibers (nonnociceptive; dorsal column-medial lemniscal pathway) or by laser-evoked potentials that are transmitted by A*δ* fibers (nociceptive) [38].

Another limitation of the present study is that we did not observe central neuropathic pain in our patients. Central neuropathic pain might occur in 50% of the MS patients [4]. Although central neuropathic pain may be present only for a short period of time [39], it may also express itself in a more continuous way [4]. Irrespective of its prevalence and expression, we might have missed central neuropathic pain in our study, as it most often occurs in the legs and feet [6]. We performed our sensory testing on the right and left hand, forearm, and upper arm. On the other hand, the presence of central neuropathic pain would not have violated our main finding, that is, a negative relationship between hypoesthesia for touch and joint position and chronic pain.

A final limitation is that we did not include an MS group *without* chronic pain in the present study. Such a control group would have been appropriate to examine the clinical and neuropathological characteristics of MS patients who do suffer from chronic pain.

## 5. Conclusion

The present study indicated a negative relationship between hypoesthesia, for light touch and joint position, and pain intensity in MS patients who suffer from chronic pain. As a consequence, the clinician should be aware of the fact that, although hypoesthesia need not per se be related to chronic pain, it *could* be an indication of chronic pain in MS patients.

## Figures and Tables

**Table 1:** Means, standard deviations, and Mann–Whitney *U* tests concerning the scores on the Beck Depression Inventory and the SCL-90 anxiety and depression scale of persons with and without multiple sclerosis (MS).

	MS patients		Controls		Mann–Whitney *U* tests	
	M	SD	M	SD	*Z*	*p*<
Beck Depression Inventory	7.42	5.04	4.75	4.39	4.02	0.001
SCL-90 depression	22.35	7.01	18.74	4.47	4.00	0.001
SCL-90 anxiety	13.89	4.73	11.89	2.47	3.06	0.003

SCL-90: symptom checklist.

**Table 2:** Means (M), standard deviations (SD), and Mann–Whitney *U* tests concerning the various sensory functions in persons with and without multiple sclerosis (MS).

	MS patients		Controls		Mann–Whitney *U* tests	
	M	SD	M	SD	*Z*	*p*<
*Dorsal column-medial lemniscal pathway*						
Light touch	1.73	0.48	1.96	0.19	3.80	0.001
Position sense	1.71	0.49	1.99	0.11	4.64	0.001

*Spinothalamic tract*						
Temperature	1.79	0.41	1.91	0.33	2.27	0.03
Pain	1.54	0.56	1.79	0.54	3.47	0.001

**Table 3:** Comorbidities that might be related to sensory disturbances in MS patients with and without sensory disturbances.

Comorbidities	MS group with sensory disturbances		MS group without sensory disturbances		Statistics	
	*n*	%	*n*	%	*χ* ^2^(1)	Fisher's exact test
Diabetes	1	3.3	2	8.0	0.58	0.59
TIA	0	0	1	4.0	1.22	0.46
Migraine	1	3.3	0	0	0.85	1.00
Epilepsy	2	6.7	0	0	1.73	0.50

MS: multiple sclerosis; TIA: transient ischemic attack.

**Table 4:** Pain experience in patients with multiple sclerosis (MS) and controls.

Pain scales	MS patients		Controls		Mann-Whitney *U* tests	
	M	SD	M	SD	Z	*p*<
NWC-A	4.76	4.09	1.05	1.98	6.75	0.001
CAS Pain Intensity	4.09	2.51	0.97	1.80	8.06	0.001
CAS Pain Affect	3.47	2.76	0.84	1.82	7.41	0.001
FPS	2.34	1.77	0.62	1.15	6.45	0.001

NWC-A: Number of Words Chosen-Affective; CAS: Colored Analogue Scale; FPS: Faces Pain Scale.

**Table 5:** Analysis of the pain medication of the patients with multiple sclerosis (MS) and controls by means of chi-square and Fisher's exact tests.

Pain medication	MS group		Controls		Statistics	
	*n*	%	*n*	%	*χ* ^2^(1)	Fisher's exact test
Baclofen	19	33.9	0	0	27.25	0.000
Paracetamol	16	28.6	1	1.5	19.07	0.000
Diclofenac	2	3.5	2	2.9	0.03	1.00
Naproxen	1	1.8	0	0	1.22	0.45
Ibuprofen	3	5.4	0	0	3.73	0.09
Cannabis	4	19.0	0	0	15.10	0.002

**Table 6:** Use of analgesics and medication that might be related to sensory disturbances (medication “sensory”) between MS patients with and without sensory disturbances.

Analgesics/medication related to sensory disturbances	MS group with sensory disturbances		MS group without sensory disturbances		Statistics	
	*n*	%	*n*	%	*χ* ^2^(1)	Fisher's exact test
*Analgesics*						
Baclofen	9	30	9	36	0.22	0.77
Paracetamol	8	26.7	7	28	0.01	1.00
Diclofenac	2	6.5	0	0	1.67	0.50
Naproxen	1	3.3	0	0	0.85	1.00
Ibuprofen	0	0	3	12	3.81	0.09
Cannabis	4	40	0	0	5.44	0.04

*Medication “sensory”*						
Sedatives	15	50	7	28	2.75	0.17
Antipsychotics	3	10	0	0	2.64	0.24
Antidepressives	5	16.7	5	20	0.10	1.00
Anxiolytics	12	40	4	16	3.81	0.08
Neurological disorders	16	53.3	8	32	2.52	0.17

**Table 7:** Hierarchical regression analyses with the domain pain intensity as a dependent variable and mood, DCML, STT, and analgesics as predictors in patients with MS (*n*=55).

Pain intensity	Beta (SE)	*t*	*p*	*F*	*df*	*p*	*R*_adj_^2^	*R* ^2^	Δ*R*^2^
*Model 1*				5.10	1.54	0.03	0.07	0.09	n/a
Mood	0.29 (0.03)	2.26	0.03						

*Model 2*				5.92	2.51	0.005	0.16	0.19	0.10
Mood	0.29 (0.03)	2.37	0.02						
DCML	−0.32 (0.64)	−2.50	0.02						

*Model 3*				3.91	3.50	0.01	0.14	0.19	n/a
Mood	0.30	2.37	0.02						
DCML	−0.34 (0.80)	−2.19	0.03						
STT	0.05 (0.81)	0.31	0.76						

DCML, dorsal column-medial lemniscal pathway; STT, spinothalamic tract.

**Table 8:** Hierarchical regression analyses with the domain pain affect as a dependent variable and mood, DCML, STT, and analgesics as predictors in patients with MS (*n*=63).

Pain affect	Beta (SE)	*t*	*p*	*F*	*df*	*p*	*R*_adj_^2^	*R* ^2^	Δ*R*^2^
*Model 1*				5.76	1.62	0.02	0.07	0.09	n/a
Mood	0.29 (0.05)	2.40	0.02						

*Model 2*				3.02	2.60	0.06	0.06	0.09	n/a
Mood	0.28 (0.05)	2.31	0.02						
DCML	−0.09 (0.99)	−0.76	0.45						

*Model 3*				1.98	3.59	0.13	0.05	0.09	n/a
Mood	0.29 (0.05)	2.30	0.03						
DCML	−0.11 (1.24)	−0.68	0.50						
STT	0.02 (1.26)	0.13	0.90						

DCML, dorsal column-medial lemniscal pathway; STT,  spinothalamic tract.
